# Influence of LAR and VAR on Para-Aminopyridine Antimalarials Targetting Haematin in Chloroquine-Resistance

**DOI:** 10.1371/journal.pone.0160091

**Published:** 2016-08-02

**Authors:** David C. Warhurst, John C. Craig, K. Saki Raheem

**Affiliations:** 1 Department of Pathogen Molecular Biology, Faculty of Infectious and Tropical Diseases and Malaria Centre, London School of Hygiene and Tropical Medicine, WC1E 7HT, London, United Kingdom; 2 Department of Pharmaceutical Chemistry, University of California San Francisco, San Francisco, CA, 94143–00446, United States of America; 3 Department of Life Sciences, Faculty of Science and Technology, University of Westminster, W1W 6UV, London, United Kingdom; Institut national de la santé et de la recherche médicale—Institut Cochin, FRANCE

## Abstract

Antimalarial chloroquine (CQ) prevents haematin detoxication when CQ-base concentrates in the acidic digestive vacuole through protonation of its *p*-aminopyridine (*p*AP) basic aromatic nitrogen and sidechain diethyl-N. CQ export through the variant vacuolar membrane export channel, PFCRT, causes CQ-resistance in *Plasmodium falciparum* but 3-methyl CQ (sontochin SC), *des*-ethyl amodiaquine (DAQ) and *bis* 4-aminoquinoline piperaquine (PQ) are still active. This is determined by changes in drug accumulation ratios in parasite lipid (LAR) and in vacuolar water (VAR). Higher LAR may facilitate drug binding to and blocking PFCRT and also aid haematin in lipid to bind drug. LAR for CQ is only 8.3; VAR is 143,482. More hydrophobic SC has LAR 143; VAR remains 68,523. Similarly DAQ with a phenol substituent has LAR of 40.8, with VAR 89,366. In PQ, basicity of each *p*AP is reduced by distal piperazine N, allowing very high LAR of 973,492, retaining VAR of 104,378. In another *bis* quinoline, dichlorquinazine (DCQ), also active but clinically unsatisfactory, each *p*AP retains basicity, being insulated by a 2-carbon chain from a proximal nitrogen of the single linking piperazine. While LAR of 15,488 is still high, the lowest estimate of VAR approaches 4.9 million. DCQ may be expected to be very highly lysosomotropic and therefore potentially hepatotoxic. In 11 *p*AP antimalarials a quadratic relationship between logLAR and logResistance Index (RI) was confirmed, while log (LAR/VAR) vs logRI for 12 was linear. Both might be used to predict the utility of structural modifications.

## Introduction

**Fig 1:**
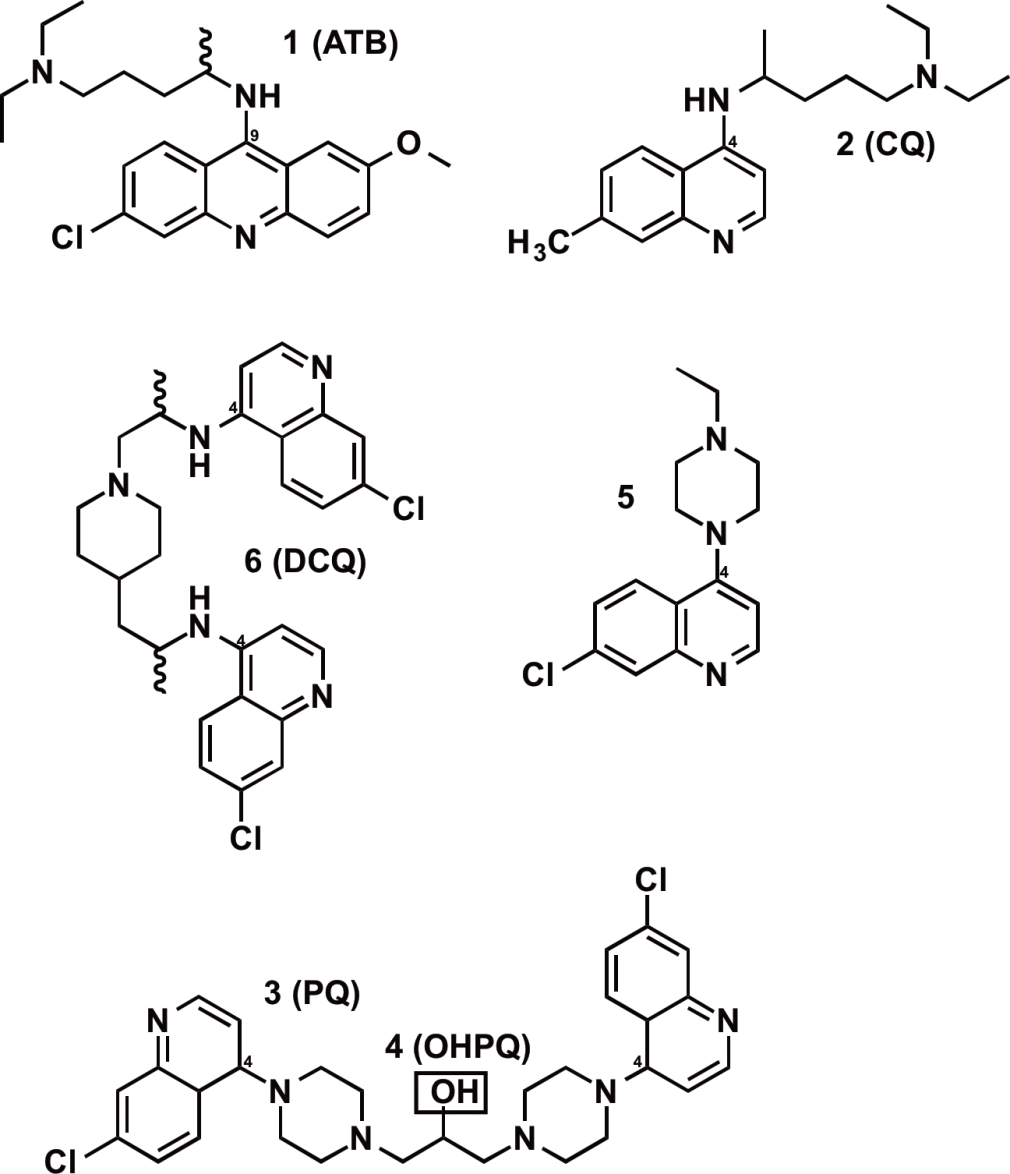
Structures of the main *p*AP compounds examined. Note outline (blue) of the *p*-aminopyridine moiety in CQ(2) and its presence in Atebrin, ATB(1) and (5). Also note 2 *p*AP moieties in each of (3), (4) and (6). Compound (5), a half–piperaquine, has low antiparasitic activity and shows 6 times less activity in the *in vitro* BIHA test than PQ (3) [14].

Early in the search for chloroquine (CQ) analogues, which could retain their antimalarial activity against the increasingly prevalent CQ-resistant strains [1] of *Plasmodium falciparum*, a group of *bis*-quinolines was synthesized [2, 3]. Structurally they were made up of two 4-amino-7-chloroquinoline units like CQ, **2**, connected at the 4-amino site by a variable aliphatic linker group containing carbon, nitrogen and sometimes oxygen atoms (Fig 1). Later, using simple alkyl linkers, in this type of *bis*-compound, branched chains or alicyclic bridges were shown to have increased activity compared to straight chains, and this was ascribed to reduced conformational mobility [4]. A similar inference may be drawn from the earlier use of two rigid cyclic diamine 1,4 piperazine moieties as the linker, in compounds such as piperaquine (PQ) 13,228 RP [3], **3** which retain high activity against CQ-resistant *P*. *falciparum* [5] (Fig 1). A similar *bis*-quinoline (**6**) also studied has as linker a single 1,4-*bis*- (propylpiperazine) unit (Fig 1). This compound, as 12,278 RP [2], was reported active against both CQ-sensitive and resistant *P*.*berghei* in mice [2, 3] though with lower efficacy on another CQ-resistant strain [6]. Named dichlorquinazine (DCQ) by Schmidt et al. its activity against CQ-resistant strains of *P*. *falciparum* in monkeys was confirmed [7], and against field isolates *in vitro*, [8] but no mechanism-directed studies have appeared since the agent was not found sufficiently effective clinically [9].

The selective toxicity of CQ (**2**) to the malaria parasite is believed to occur within the infected erythrocyte, and specifically in the digestive vacuole of the parasite through binding to haematin and preventing its detoxication to crystalline β-haematin (haemozoin) [10–12]. The entry of a basic drug through lipid membranes and its distribution into the aqueous compartments of an infected erythrocyte is determined by the lipid-water partition coefficient- (expressed as Log P), as modified by pH through the ionization constants (pKa) of the basic centre (s) of the drug to give the pH-modified coefficient Log D [13]. This raised the question whether these two *bis* quinolines (**3** and **6**) of similar molecular weight (535 for **3** vs. 523 for **6**) may owe their biological properties to identical physicochemical parameters in respect of their acid-base character and lipid solubility.

We therefore measured the pKa and log P values of DCQ, in comparison with those of PQ and CQ. In addition, the activity of DCQ in the prevention of haematin detoxication by dimerization to β-haematin (β-haematin inhibition assay: βHIA) has been determined, as was earlier done for CQ, PQ, OHPQ and cpd 5 (a fragment of PQ) [13].

## Material and Methods

Dichlorquinazine **6**, 12,278 RP, was supplied by Rhone-Poulenc [14]. Since DCQ is centro-symmetric and contains two chiral centres, it can exist in the optically active R,R or S,S form {mp 250–251°C, [α]D + or -382° (MeOH)}, or in the non-resolvable meso (internally compensated or RS) form with mp 270–271°C, [α]D + 0°. The present test material has mp 249–250°C and [α]D + 0°, and was identified from its properties as a 1:1 mixture of the R,S and meso forms (the expected synthesis product). (mp reported: 250°C) [14–15]. Analysis predicted from composition C_28_H_32_CI_2_N_6_, would be: C, 64.24; H, 6.19; N, 16.06: found: C 64.4; H 6.30; N 15.93%. Samples of PQ **3** and OHPQ **4** were obtained from WHO (Dr. Piero Olliaro and Dr. Alan Shapiro) as the tetraphosphate tetrahydrate and CQ **2** was supplied as the racemic disphosphate by Sigma, Cpd.5, **5** was synthesized as reported in [13].

### Physicochemical

The partition coefficient (log P) is measured for the un-ionized drug. For partially ionized compounds the partition coefficient at any fixed pH is called the distribution coefficient on the assumption that only the un-ionized base partitions from the aqueous to the lipid phase [15]. At any given pH, logD is then obtained from Eq 1 [16–17]. The ionized basic centres of CQ **2** are (1) a resonance-stabilized aromatic amidinium ion made up of the protonated N-1 of the quinoline and an amino group attached by a single bond to C-4 of the same ring, and (2) the distal aliphatic protonated N. Thus Eq 1 needs modification to Eq 2. PQ **3**, and DCQ **6** are centro-symmetric and have two of each type of basic centre. Initial protonation at one basic centre may affect the other via a through-space electrostatic effect of the now positively charged N ion on the other (uncharged) N atom, causing a slight reduction of the basicity of the second basic centre by inhibiting its protonation. The existence of 4 separate pKa values for PQ and DCQ is therefore possible, and to provide for the contribution from all ionized species to the log D, Eq 2 is modified to Eq 3. (see below).

$$\log D=-\log P-\log[1+10^{(pKa-pH}]$$ (1)

$$\log D=-\log P-\log[1+10^{\left(pKa1-pH\right)}+10^{\left(pKa1+pKa2-2pH\right)}]$$ (2)

$$LAR=anti\log\;\log D7.4,andVAR=[anti\log(\log D7.4-\log D^{pHVac})$$ (3)

It should be noted that in Eqs 2 and 3, pKa1 ≥ pKa2 ≥ pKa3 ≥ pKa4.

Eqs 1 and 2 and 3 make it possible to calculate log D from observed log P, pKa and pH [17]. As well as experimentally measuring log P, it can be estimated using the Clog P program developed by Hansch and Leo [16] and various modifications such as the ACD suite (www.acdlabs.com/products/percepta/physchem/admetox/). Dissociation constants and partition coefficients were experimentally determined by Robertson Microlit Laboratories (Madison NJ USA) at 25°C using the Sirius GLpK automated computerized potentiometric system [18] which is capable of resolving ionization constants of multiprotic substances. Titration employed water containing 0.15M KCl (representing a physiological concentration of positive and negative counter-ions) in an argon atmosphere. The pKa values were determined in triplicate with a SE of ± 0.20. For PQ **3** and DCQ **6** the base precipitated above pH 7.0. The titrations were therefore carried out with methanol as a co-solvent, using 5 different ratios of methanol to water. The aqueous pKa was determined by extrapolation to 0% methanol [19] using the method of Yashuda-Shedlovsky which gives a linear fit. LogD values at the physiological pH of 7.4 and at pH 4.8 as previously used [13], were calculated from Eq 2 or Eq 3. Partition coefficients between octanol and water were measured by dual-phase potentiometric titration using graded amounts of water-saturated n-octanol. Titrant addition was carried out with vigorous stirring of the assay solution. Three different ratios of octanol/water were employed for each compound. The log P values were obtained from the difference between the aqueous pKa of the species and the apparent pKa determined from the dual phase titration. Measurements were carried out in triplicate with a S.E of ± 0.40. The potentiometric method was validated by comparison with results obtained by the standard shake-flask technique [19].

**Fig 2:**
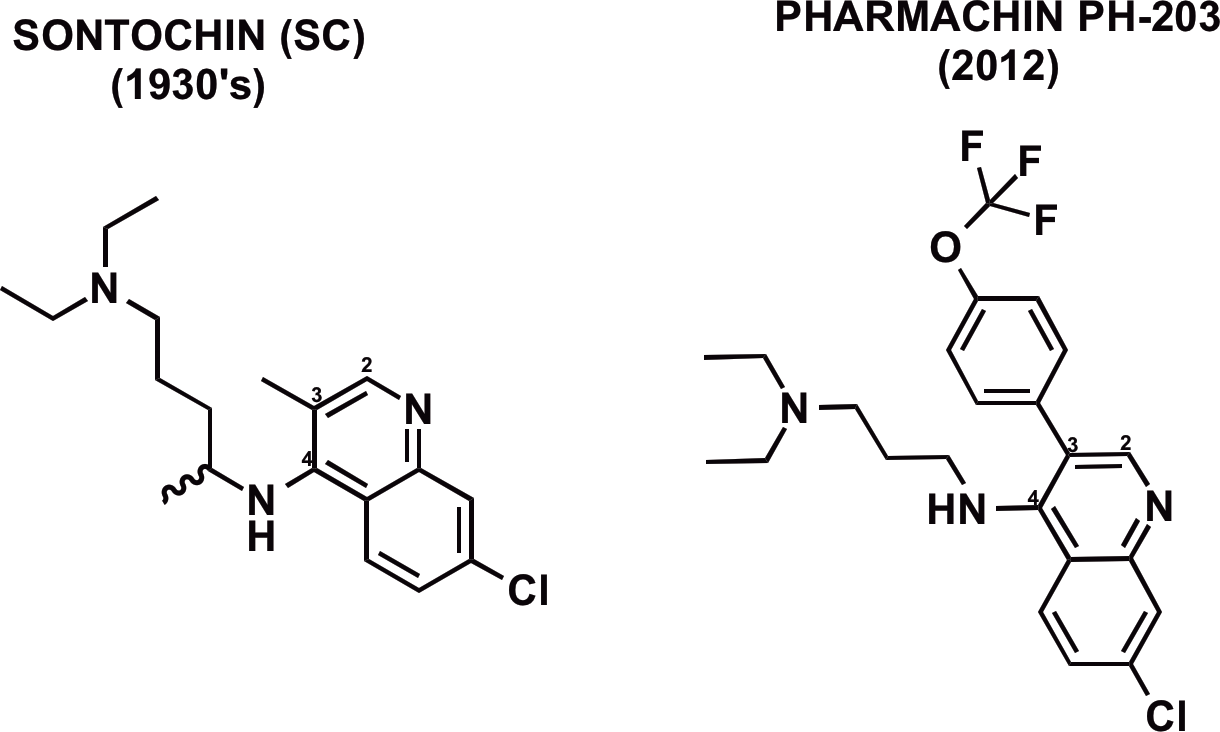
Structures of the German CQ (resochin) replacement sontochin (SC) and PH-203, a highly active *p*AP recently developed from SC [20].

For a direct comparison between CQ, synthesized in 1930s Germany, and its replacement sontochin (SC) its less toxic 3-methyl derivative (Fig 2), it was important to compare measured values for both. We did not have access to SC and our first estimates were made entirely by calculation and resulted in logD (7.4) of 2.024 and log D (4.8) of -2.268. Fortunately Irvin and Irvin (1947) [20] had compared the pKa values of CQ and SC directly by titration using a technique closely similar to current practise and obtained pKa values for CQ of pKa1: 10.16 and pKa2: 8.08 (our measured values had been 10.18 and 8.38). Their measurements for SC were 10.15 and 7.28, and were used in preference to those we calculated using the ACD suite, resulting in log D(7.4) of 2.15 and logD(4.8) of -2.68 (**see Table 1 and S1 Table**). The calculated Log P values from ACD (based on CLog P [16]) are reliable when compared with measurement.

**Table 1:** Physicochemical and other parameters for the compounds studied.

											antilog	LAR/VAR					
								logD4.8	logD7.4	Log CQ RI	logD7.4	Antilog		D7.4-D4.8	BHIA	BHIA	BHIA
Drug	logP	pKa1	pKa2	pKa3	pKa4	pH	logD	(LAR/VAR)	LOGLAR	(res/sens)	LAR	logD4.8	VAR	logVAR	IC50 mM	SE	n
CQ	4.72	10.18	8.38	-20	-20	7.4	0.91668						1434882	5.156796	1.3	0.11	8
CQ	4.72	10.18	8.38	-20	-20	4.8	-4.24011	-4.2401	0.91668	1.149	8.25434	5.75E-05					
PQ	6.11	6.88	6.24	5.72	5.39	7.4	5.98833						104378.2	5.01861	0.62	0.05	6
PQ	6.11	6.88	6.24	5.72	5.39	4.8	0.96972	0.96972	5.98833	0.39	973492	9.3266					
OHPQ	5.67	6.6	6.41	5.39	4.83	7.4	5.60001						19874.33	4.298293	0.58	0.09	6
OHPQ	5.67	6.6	6.41	5.39	4.83	4.8	1.30172	1.30172	5.60012	0.176	398118	20.032					
DCQ	6.1	8.71	8.34	7.36	5.9	7.4	4.19						48897788	6.69	0.61	0.09	7
DCQ	6.1	8.71	8.34	7.36	5.9	4.8	-2.5	-2.5	4.19	0.176	15488.2	0.0032					
DCQa	6.1	8.71	8.34	7.36	5.9	7.4	3.53586						3.79E+08	8.579145	0.61	0.09	7
DCQa	6.1	8.71	8.34	7.36	5.9	4.8	-5.04328	-5.0433	3.53586	0.176	3434.48	9.05E-06					
5	3.48	7.92	5.54	-20	-20	7.4	2.84081						1965.476	3.293468	3.35	0.33	9
5	3.48	7.92	5.54	-20	-20	4.8	-0.45266	-0.4527	2.84081	1.308	693.123	0.3526					
HCQ	3.835	9.66	8.27	-20	-20	7.4	0.64976						139607.1	5.144907			
HCQ	3.835	9.66	8.27	-20	-20	4.8	-4.49515	-4.4952	0.64976	1.898	4.46437	3.20E-05					
DECQ	4.35	10.96	8.4	-20	-20	7.4	-0.2514						144113.8	5.158706			
DECQ	4.35	10.96	8.4	-20	-20	4.8	-5.41011	-5.4101	-0.2514	1.564	0.56053	3.89E-06					
DAQ	3.31	8.72	7.53	-20	-20	7.4	1.61036						89365.84	4.951172			
DAQ	3.31	8.72	7.53	-20	-20	4.8	-3.34081	-3.3408	1.61034	0.732	40.7721	0.0005					
AQ	4.26	8.66	7.05	-20	-20	7.4	2.82344						47410.07	4.675871			
AQ	4.26	8.66	7.05	-20	-20	4.8	-1.85244	-1.8524	2.82344	0.297	665.94	0.014					
ATB	4.85	10.47	7.12	-20	-20	7.4	1.59654						54779.28	4.738616			
ATB	4.85	10.47	7.12	-20	-20	4.8	-3.14207	-3.1421	1.59654	0.682	39,4951	0.0007					
SC	5.15	10.15	7.28	-20	-20	7.4	2.1544						68522.82	4.835835			
SC	5.15	10.15	7.28	-20	-20	4.8	-2.68144	-2.6814	2.1544	0.376	142.92	0.0021					
PH203	6.45	10.29	5.57	-20	-20	7.4	3.55307						2698.94	3.41193			
PH203	6.45	10.29	5.57	-20	-20	4.8	0.12188	0.12188	3.55307	0.193	3573.32	1.324					

### Molecular Modelling

Earlier we found molecular modelling using AM1 [13] helpful in interpreting stoichiometry of interactions with haematin [13], and also to test expectations of resonance within the para (4)-aminopyridine ring and the para-aminopyridinium ion formed on protonation of the aromatic ring N1.

Structures and energies were calculated in vacuo (in view of our interest in lipid association) in the Restricted Hartree- Foch (RHF) state, singly excited, with 15 occupied and 15 unoccupied orbitals).

## Results and Discussion

The original question was whether two *bis* quinolines (**3** and **6**) of similar molecular weight (535 for 3 vs. 523 for 6) might owe their biological properties to identical physicochemical parameters in respect of their acid-base character and lipid solubility. This is apparently true only to a certain extent. The measured dissociation constants and log P, and the calculated and measured log D values at pH 7.4 and 4.8 for compounds **1–6** are shown in Table 1. It is seen that in **3**, **4** and **6** stepwise protonation of the two quinoline ring N atoms can occur, resulting in a small reduction of pKa for the second quinoline N due to the slight base-weakening effect of the first protonated N. This is shown in DCQ **6** by the pKa1, and pKa2 values of 8.71 and 8.34 for the two aromatic para-aminopyridinium ions (**Table 1 and S1 Table**), in good agreement with the value for CQ **2** (8.38). While the pKa for N-1 in piperazine itself is 9.81, alkyl substitution of a secondary N to become a tertiary N lowers its basic strength by ca. 1 pKa unit, e.g. N (2-aminoethyl) piperazine has pKa of 8.51, and further alkyl substitution at N-4 brings it to ca. 7.5, in agreement with the experimental 7.36 found for pKa3. In the case of piperazine, the pKa of N-4 when N-1 is protonated is 5.5, i.e. prior protonation of N-1 here causes a much larger base-weakening effect due to the close proximity of the two basic centres. This is in good agreement with the observed value of 5.9 for this pKa for DCQ **6**.

The results in Table 1 reveal that while the basic character of PQ **3** is affected to a major extent (100-fold reduction in basic strength) by each piperazine-N-1 being also the 4-amino N of each quinoline, in DCQ **6** each secondary N-4 para-aminopyridinium system of CQ remains intact, with no change in pKa1 or pKa2 from that in CQ. On the other hand the more basic terminal diethylamino group (pKa 10.18 in CQ **2**) has been replaced in DCQ **6** by the much weaker basic N-1 and N-4 of piperazine (pKa 7.36 and 5.90). This change in pKa characteristics is indeed reflected in the observed values of logP (**Table 1**).

While the expected high lipophilicity of PQ **3** (log P: 6.11) is replicated in DCQ **6** (6.10), it should be noted that log P reflects only the partition behaviour for the compound in the free base state. At physiological pH values, the two compounds behave differently. For PQ **3** at pH 7.4, the measured logD of 5.99, is essentially unchanged from the log P value 6.11. However, because mean pKa for DCQ **6** (ca. 8.5) is two units higher than that for PQ 3)(ca. 6.5), measured logD (pH 7.4) for DCQ **6** drops by 2 logs to 4.19 (calc. 3.54).

Our studies [13] of the antiplasmodial activity of PQ **3** suggested that retention of activity against CQ-resistant strains of *P*. *falciparum* was linked to its enhanced lipophilic properties indicating optimal membrane transfer at pH 7.4 into the lipid phase, together with a high degree of accumulation in the acidic vacuolar water content, where its haematin interaction capability (per quinoline *p*AP unit) equals that of CQ **2**. While haematin in the parasite digestive vacuole is a target of both PQ and CQ, a high concentration of drug in the digestive vacuole water appears not to be enough for activity in CQ-resistance, since PQ **6** (and amodiaquine/ desethylamodiaquine) clearly concentrate within membrane or intravacuolar lipid droplets in the infected erythrocyte and may also hydrophobically bind inside the export channel and inhibit transport [21–25].

To some extent, the influence of the ionization behaviour of DCQ **6** on its intracellular distribution similarly determines its unusual biological properties. At physiological pH, with a mean pKa of 8.55, the compound exists almost totally (>95%) as the mono-protonated quinolinium salt (the same is true for CQ **2** with a pKa of 8.38). In addition, the proximal N-1 piperazine nitrogen in DCQ **6** (pKa 7.36) is 50% ionized at pH 7.4 (the distal N-4 nitrogen with pKa 5.90 remains >95% in the free base form). The resultant loss in lipophilicity for DCQ **6** between log P and log D 7.4 is reflected in a decrease of 2 log units. (For CQ **2**, its side-chain N with pKa 10.18 is totally protonated at pH 7.4 and no longer makes a lipophilic contribution compared with log P, resulting in a drop of almost 4 log units).

At pH 4.8, all basic centres of DCQ **6** are essentially totally ionized (100%, 100%, 83% respectively). This is reflected in the drop of observed log D (pH 4.8) to -2.5. Differences between the effect of drugs against CQ-resistant mutants may depend on the relationship between LAR and VAR. Given an equal inhibitory effect on haematin dimerization and formation of hemozoin (β-hematin), value of VAR determines the effect on CQ-sensitive parasites (**Table 1 and S1 Table**). However, as the hydrophobicity and accessibility of the CRT channel in the CQ-R parasites increases, the interaction of the drug with the lining residues of the channel appears to be determined by the LAR [21, 22], and so a high LAR is preferable. Comparing the VAR values of PQ **3** and DCQ **6**, that of the latter drug is very much higher. Yet for activity in CQ-resistant parasites high LAR values are apparently more important [13].

Although observed and calculated log D (pH 7.4) values for PQ **3** and DCQ **6** studied by both Robertson Microlit and using Eq 3 are closely similar, at pH 4.8 there is over 2 orders of magnitude difference between observed and calculated log D (pH4.8) values for DCQ **6** (-2.5 and -5.043) not seen for the less basic PQ **3** (0.97 and 1.02). This may reflect a difference in the algorithm [16] used by Robertson Microlit Laboratories to estimate the acidic log D values (in the protocol using the Sirius GlpKa machine), and is in contrast to Eqs (1–3) which we normally use to obtain log D from log P [13]. However in view of the mixed population of DCQ structures contained in the test material, it could also reflect intermolecular association effects or solubility problems not detected by our calculation.

On comparing log (LAR/VAR) with log D_pH4.8_, we realized that these values are identical. This is true for any vacuolar pH, because[13]:

$$LAR=anti\log\log D_{7.4},andVAR=[anti\log(\log D_{7.4}-\log D_{pHVac})].$$

Given suitably accurate values for the Log D value of a drug at pH 7.4 (log D _(pH 7.4)_) and at the pH of water in the digestive vacuole of the malaria parasite (log D_(pHVAC)_), it is possible to estimate the lipid accumulation ratio (LAR) of the drug:

$$LAR=anti\log\;\log D_{\left(pH7.4\right)}$$

and its accumulation into vacuolar water (VAR).

$$VAR=anti\log[(\log D_{\left(pH7.4\right)})-(\log D_{\left(pHVAC\right)}].$$

It also follows from this that the value of log(*LAR*/*VAR*) = (log*D*_(*pHVAC*)_)

### Molecular Modelling:

In **Table 2**. based on the AM1 calculation [13], we notice the similar short distance between N-1 and 4-amino N atoms in the diprotonated form, of CQ2H+ **2** and DCQ2H+ **6**. This indicates a marked resonance effect in both, which would predict their similar observed N-1 pKa values contrasting with the lower N-1 pKa values for PQ2H+. We found molecular modelling using AM1 [24] helpful in interpreting stoichiometry of interactions with haematin earlier [13], and here this has been used to test expectations of resonance between the para-amino substituent of the pyridine ring and the pyridinium ion formed on protonation of the aromatic ring N. The para-aminopyridinium ion is found in each quinoline (CQ, PQ, OHPQ and DCQ). It is located in the acridine ring for ATB **1**. We have been prevented by the multiple protonated state of pyronaridine, a relative of ATB and AQ, from making predictions about the physico-chemistry of that drug, but we expect similar conclusions on relationship of VAR and LAR to activity to hold.

**Table 2:** AM1 energies. Note reduced distance between N-1 and 4-amino N atoms in all the diprotonated forms and increased separation of quinolines N1 in tetraprotonated forms of DCQ.

AMI (rms 0.001)	R&S	R&S	R&S	PQ	PQ2H+	PQ4+	R-DCQ,S-	R-DCQ,S-	R-DCQ,S-	*MESO-*	*MESO-*	*MESO-*
	CQ	CQ1H+	CQ2H+		(N-1)		DCQ	DCQ (2H+)	DCQ (4H+)	RS-DCQ	RS-DCQ N1-2H+	RS-DCQ 4H+
Total energy kcal/mol	-84345	-84513	-84650	-141613	-141941	-142025	-138676	-139012	-139065	-138675	-139010	-139071
HOF kcal/mol	23.65	170.2	347.9	126.7	428.8	974	106.2	400.7	977.4	107.9	402.1	971.3
distance between quinolines N-1	NA	NA	NA	17.65Å	18.29Å	18.3Å	15.01Å	16.43Å	**16.91Å**	11.93Å	15.99Å	**17.29Å**
bridging sc N-N	NA	NA	NA	4.985Å	4.965Å	5.073Å	2.986Å	2.985Å	3.036Å	2.952Å	2.983Å	3.019Å
quinoline N-1	7.893Å	7.999Å	8.991Å	6.526Å	6.827Å	6.83Å	6.038Å	6.992Å	7.0Å	7.585Å	6.806Å	7.662Å
to terminal sc-N				6.52Å	6.819Å	6.837Å	6.582Å	6.836Å	7.032Å	6.774Å	7.534Å	7.571Å
separation of N-1	4.255 Å	4.258 Å	**4.196** Å	4.280	**4.202**	4.231	4.282	**4.187**	4.234	4.260	**4.198**	4.221
and 4-amino N				4.280	**4.200**	4.231	4.252	**4.189**	4.222	4.249	**4.186**	4.210

In addition to the resonance aspect for the drugs we have analysed, it is notable how the increased flexibility of the meso form (RS) of DCQ base **6** allows a greater distance between the quinoline rings on protonation compared with the RR and SS forms (**Table 2**) probably resulting in lesser mutual supression of protonation for the meso component.

### Graphical approaches

**Fig 3:**
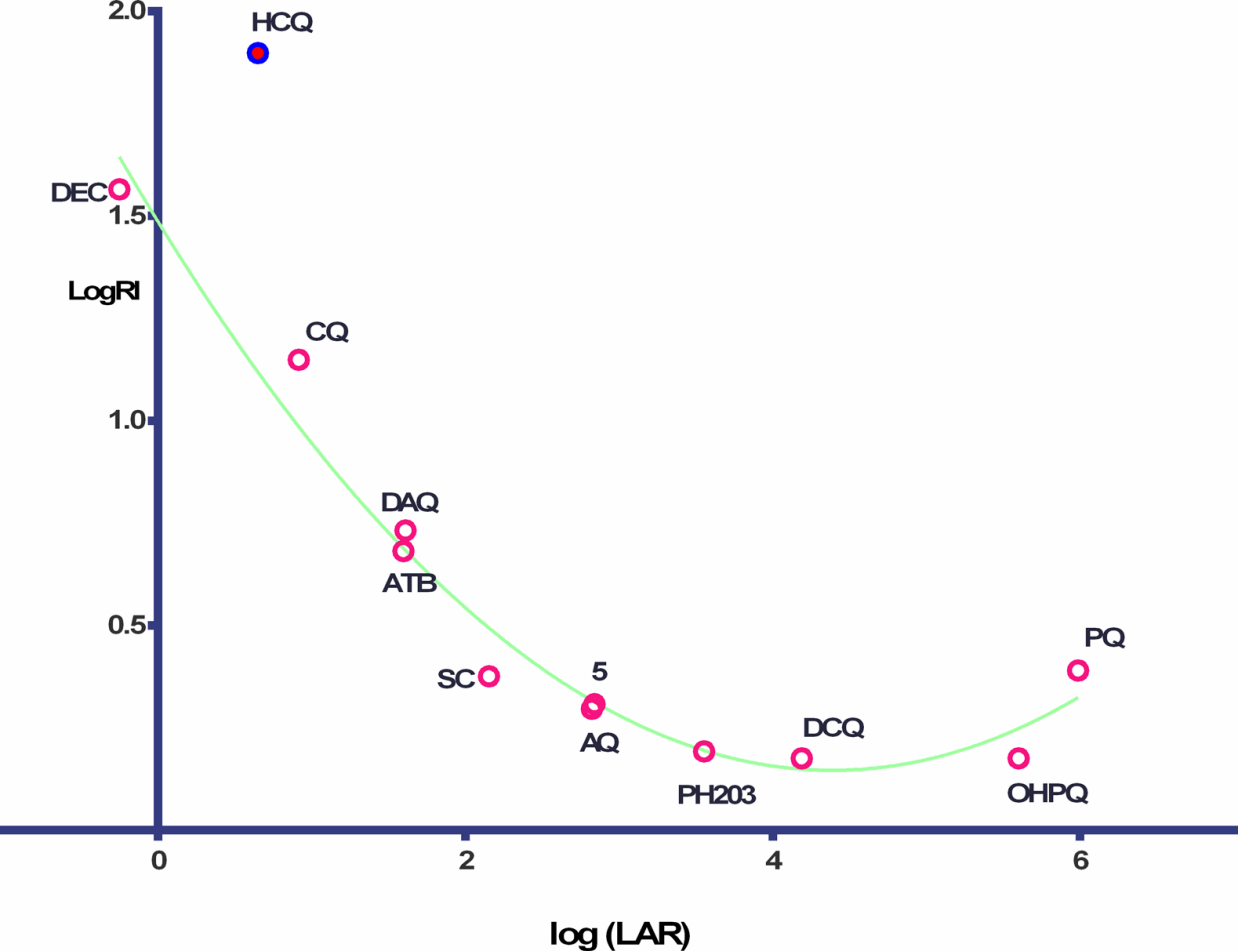
2^nd^ order polynomial for logRI (y) on Log LAR(x) for 11 *p*-aminopyridines and one outlier.

We had originally observed for 8 structures an inverse, 2^nd^ order polynomial relationship (quadratic) between log LAR and log Resistance Index (log RI): y = 0.072*x^2^-0.6675x + 1.7174, R^2^ was 0.8735) [13] and for the 12 structures examined here which include the less toxic early German replacement for chloroquine, Sontochin (SC) (clinically tested on the North African battlefield, captured by the allies, and transferred to the US) and another 3-substituted CQ-related structure, PH-203 (in development [20]) a similar quadratic equation (with HCQ classed as an outlier) was observed (Fig 3).

$$y=0.07008\;*\;X^{2}-0.6145x+1.489,$$

$$R^{2}=0.9706$$

**Fig 4:**
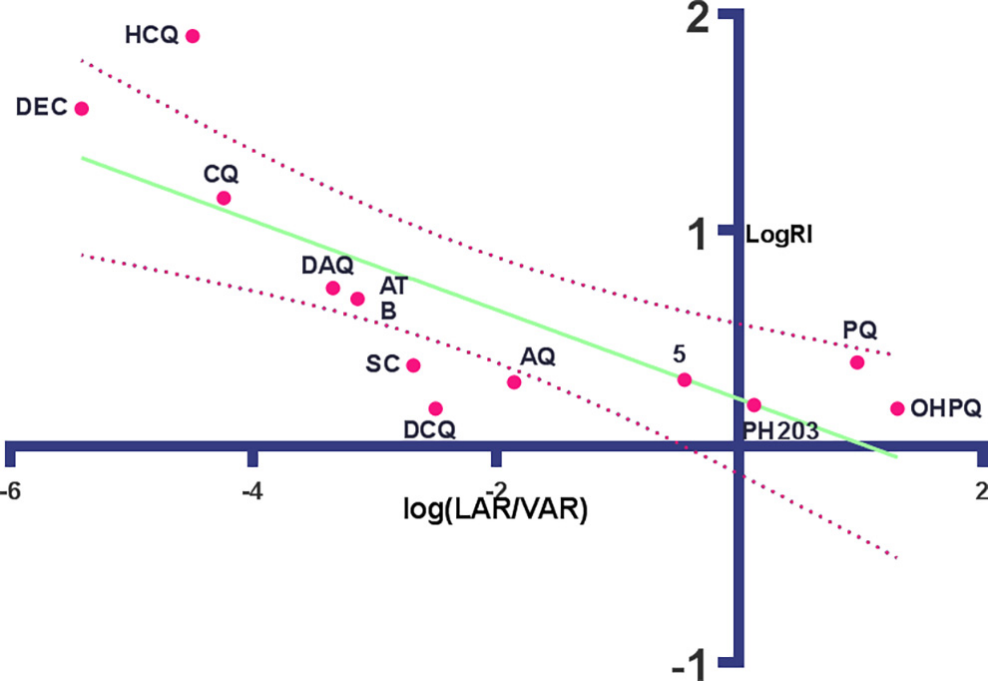
Linear regression of log Resistance Index, (y) vs log (LAR/VAR), (x). (12 *p*-amino pyridines).

Log LAR and log VAR clearly both have an influence on activity and in **Fig 4**, we detect an inverse linear relationship between log (LAR/VAR) or log D_(pHVAC)_ and log RI.

$$\begin{array}{l} y=-0.2178x+0.1774\\ R^{2}=0.6315\\ P=0.002 \end{array}$$

When the value of log (LAR/VAR) (or (log D_(pHVAC)_) increases, log Resistance index, RI, approaches zero,. This indicates that both LAR and VAR are important, not surprising since a high drug concentration in the vacuolar water is important for inhibition of haematin dimerization, and in resistance the CQ-exporter PfCRT is blocked and transport inhibited by the hydrophobic concentrated drug from the vacuolar water. The equation for this linear regression, might therefore be used to predict log RI for a variety of *p*AP structural variants, where the only physicochemical data available are the 2 log D values.

In view of the very high VAR values we predict for DCQ, this is in agreement with the high liver localization noted at the start of its history [2, 3] and it may be regarded as fortunate that such a highly lysosomotropic agent was not tested clinically on any scale.

## Supporting Information

S1 Table Physicochemical and other parameters for the compounds studied. (XLSX)
