# Correction: Influence of LAR and VAR on Para-Aminopyridine Antimalarials Targetting Haematin in Chloroquine-Resistance

**DOI:** 10.1371/journal.pone.0162142

**Published:** 2016-08-30

**Authors:** David C. Warhurst, John C. Craig, K. Saki Raheem

There are errors in the published article.

Fig 1 does not show the enantiomeric bond from 4-amino to methyl. In the caption for Fig 1, the reference given is incorrect. The correct reference is [13]. Please see the correct Fig 1 and its caption here.

**Fig 1:**
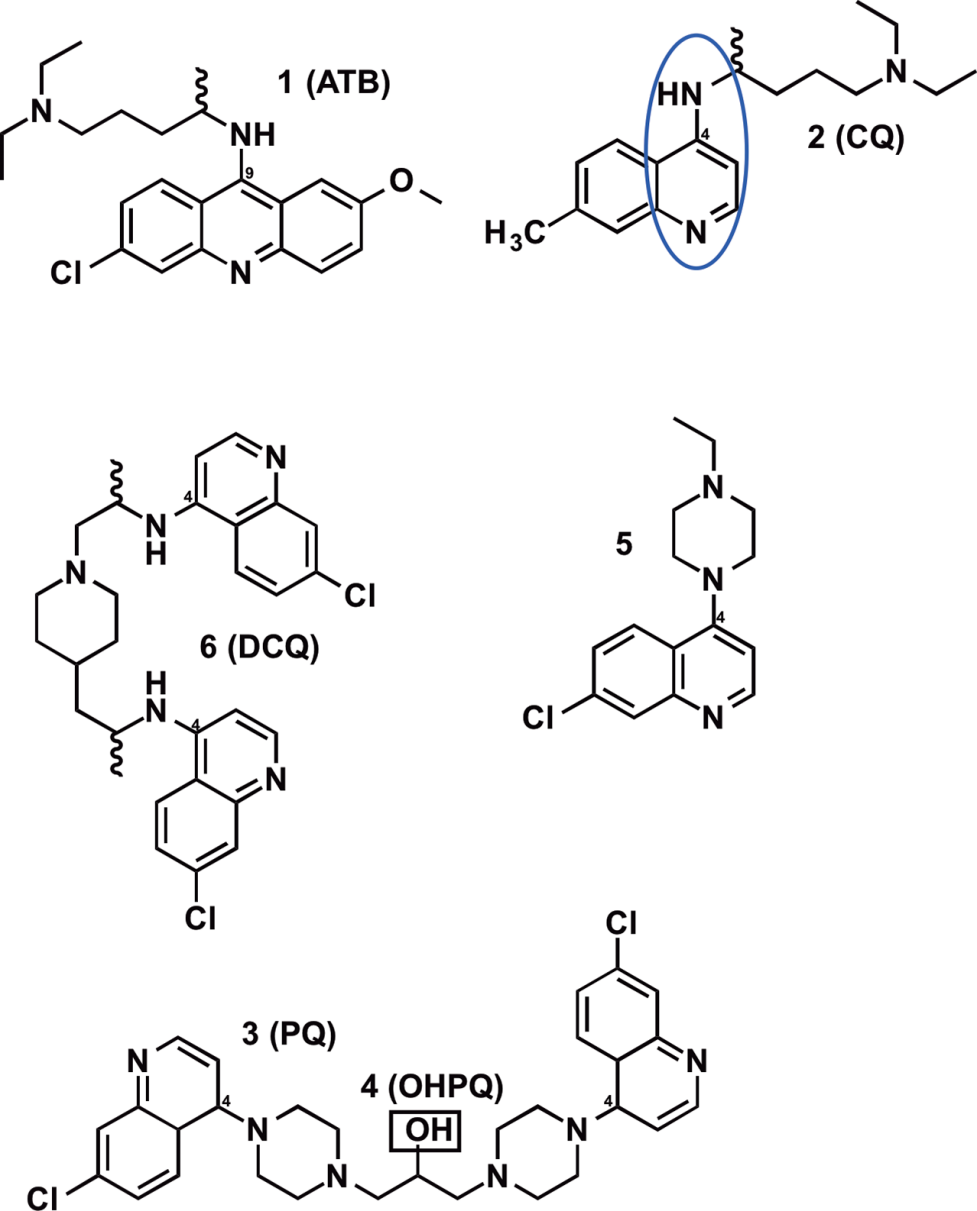
Structures of the main *p*AP compounds examined. Note outline (blue) of the *p*-aminopyridine moiety in CQ(2) and its presence in Atebrin, ATB(1) and (5). Also note 2 *p*AP moieties in each of (3), (4) and (6). Compound (5), a half–piperaquine, has low antiparasitic activity and shows 6 times less activity in the *in vitro* BIHA test than PQ (3) [13].

In the Physiochemical section of the Material and Methods, the third equation appears incorrectly. The correct equation is:

$$logD=logP-log[1+10^{\left(pKa1-pH\right)}+10^{\left(pKa1+pKa2-2pH\right)}+10^{\left(pKa1+pKa2+pKa3-3pH\right)}+10^{\left(pKa1+pKa2+pKa3+pKa4-4pH\right)}]$$ (3)

In the Molecular Modelling sections of the Material and Methods and the Results and Discussion, the reference given is incorrect. The reference should be [25].

The references that appear at the end of the fourth paragraph in the Results and Discussion are incorrect. The correct references are [21–24].

In the last sentence of the Graphical Approaches section of the Results and Discussion the incorrect reference appears. The correct reference is [21].

There are errors in Table 1. In the LogCQRI column the value for cpd5 should be 0.308, not 1.308. In the VAR column the value for CQ should be 143482, not 1434882. The value for the first DCQ should be 4897788, not 48897788.

**Table 1:** Physicochemical and other parameters for the compounds studied.

											antilog	LAR/VAR					
								logD4.8	logD7.4	Log CQ RI	logD7.4	Antilog		D7.4-D4.8	BHIA	BHIA	BHIA
Drug	logP	pKa1	pKa2	pKa3	pKa4	pH	logD	(LAR/VAR)	LOGLAR	(res/sens)	LAR	logD4.8	VAR	logVAR	IC50 mM	SE	n
CQ	4.72	10.18	8.38	-20	-20	7.4	0.91668						143482	5.156796	1.3	0.11	8
CQ	4.72	10.18	8.38	-20	-20	4.8	-4.24011	-4.2401	0.91668	1.149	8.25434	5.75E-05					
PQ	6.11	6.88	6.24	5.72	5.39	7.4	5.98833						104378.2	5.01861	0.62	0.05	6
PQ	6.11	6.88	6.24	5.72	5.39	4.8	0.96972	0.96972	5.98833	0.39	973492	9.3266					
OHPQ	5.67	6.6	6.41	5.39	4.83	7.4	5.60001						19874.33	4.298293	0.58	0.09	6
OHPQ	5.67	6.6	6.41	5.39	4.83	4.8	1.30172	1.30172	5.60012	0.176	398118	20.032					
DCQ	6.1	8.71	8.34	7.36	5.9	7.4	4.19						4897788	6.69	0.61	0.09	7
DCQ	6.1	8.71	8.34	7.36	5.9	4.8	-2.5	-2.5	4.19	0.176	15488.2	0.0032					
DCQa	6.1	8.71	8.34	7.36	5.9	7.4	3.53586						3.79E+08	8.579145	0.61	0.09	7
DCQa	6.1	8.71	8.34	7.36	5.9	4.8	-5.04328	-5.0433	3.53586	0.176	3434.48	9.05E-06					
5	3.48	7.92	5.54	-20	-20	7.4	2.84081						1965.476	3.293468	3.35	0.33	9
5	3.48	7.92	5.54	-20	-20	4.8	-0.45266	-0.4527	2.84081	0.308	693.123	0.3526					
HCQ	3.835	9.66	8.27	-20	-20	7.4	0.64976						139607.1	5.144907			
HCQ	3.835	9.66	8.27	-20	-20	4.8	-4.49515	-4.4952	0.64976	1.898	4.46437	3.20E-05					
DECQ	4.35	10.96	8.4	-20	-20	7.4	-0.2514						144113.8	5.158706			
DECQ	4.35	10.96	8.4	-20	-20	4.8	-5.41011	-5.4101	-0.2514	1.564	0.56053	3.89E-06					
DAQ	3.31	8.72	7.53	-20	-20	7.4	1.61036						89365.84	4.951172			
DAQ	3.31	8.72	7.53	-20	-20	4.8	-3.34081	-3.3408	1.61034	0.732	40.7721	0.0005					
AQ	4.26	8.66	7.05	-20	-20	7.4	2.82344						47410.07	4.675871			
AQ	4.26	8.66	7.05	-20	-20	4.8	-1.85244	-1.8524	2.82344	0.297	665.94	0.014					
ATB	4.85	10.47	7.12	-20	-20	7.4	1.59654						54779.28	4.738616			
ATB	4.85	10.47	7.12	-20	-20	4.8	-3.14207	-3.1421	1.59654	0.682	39,4951	0.0007					
SC	5.15	10.15	7.28	-20	-20	7.4	2.1544						68522.82	4.835835			
SC	5.15	10.15	7.28	-20	-20	4.8	-2.68144	-2.6814	2.1544	0.376	142.92	0.0021					
PH203	6.45	10.29	5.57	-20	-20	7.4	3.55307						2698.94	3.41193			
PH203	6.45	10.29	5.57	-20	-20	4.8	0.12188	0.12188	3.55307	0.193	3573.32	1.324					
